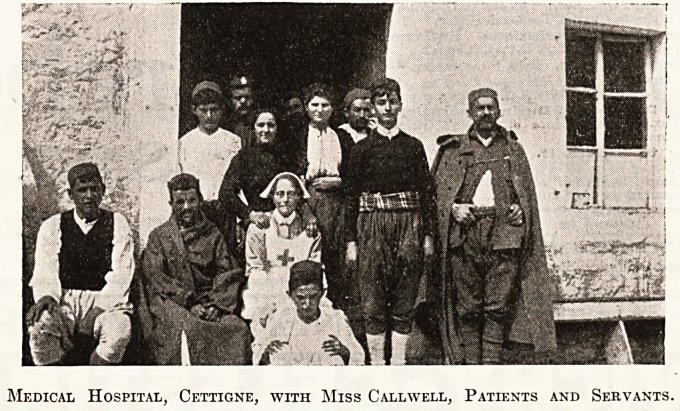# British Workers in Foreign Countries

**Published:** 1913-08-30

**Authors:** 


### BRITISH WORKERS in FOREIGN COUNTRIES.

### WITH THE WOUNDED IN MONTENEGRO—II.*

By personal application I used occasionally to get delicacies from the Red Cross Committee; these were hoarded up, and as I had all the enteric cases (and was therefore "tabû" to the Royal princesses, who feared the word fever and consequently never brought good things to my hospital as they did to the others) I found I myself had to become importunate. Charitable societies from all over Europe sent clothing, and I was amused one day on requisitioning 20 pairs of drawers to receive 20 petticoats tied up with a label still on the bundle "From a sewing-party in Montreux."

### My Hospital.

When we arrived on February 16 we were warmly welcomed by the foreign doctors. With the exception of a few more or less trained Russian nurses and two nuns from Ragusa there were only a few volunteers to help them. We three were quickly set to work, Miss R. taking charge of one floor in the Danilo Hospital which was full of serious cases, and where the Matron was delighted to have her, as her own staff was constantly depleted by requests for her nurses to be sent to various emergency hospitals. Miss A. started work in the Government building and later took charge of a temporary hospital erected in the grounds of the Royal Palace at Rieka. I took over a dilapidated schoolhouse which before long proved all too small for medical cases, which towards the end of the campaign increased in numbers owing to the hardships endured before Scutari. The hardy mountaineers were struck down in hundreds by rheumatism, bronchitis and pneumonia.

Most of the enteric cases went direct from the front to Podgoritsa, where an Italian Mission took charge of them. The water supply in Cettigne is pure, being drawn from artesian wells; my patients came direct from the trenches and were passed on to me from the surgical wards as soon as the symptoms were observed. Owing to their injuries and the numbers to be dealt with they did not usually reach me till they were half through, and sometimes practically over, the illness.

### Curious Ideas of Cleanliness.

Disinfectants there were none, and owing to the dirty habits of the people the yard was in an indescribable state of filth, but after a week of indignant appeals and prophecies of a probable epidemic I got good supplies of chloride of lime and carbolic. This was the more necessary as the house being lower than the yard heavy rain produced a cataract, which "seven maids with seven mops" could not have kept back from flooding the passage paved with large blocks of stone, with resultant crevices. The sweepings of the wards for three months had been also carefully stored in this passage, but after I had it removed by Turkish prisoners and the authorities sent some cartloads of gravel for the yard, I felt more easy in my mind. I had two wardmaids—one as beautiful as a Greek goddess—who had the most rudimentary ideas as to cleanliness. Their only way of washing the floor was to throw buckets of water over it and then sweep it out of the door; this left the uneven boards wet for days, and there were several muddy puddles as there were large holes where the flooring had rotted away. However, brushes and soap were procured, and before I left a weekly scrubbing had done wonders, and the women soon became proud of their respective wards. I scrubbed the window-paint and nailed up muslin blinds to hinder the passers-by seeing all the operations proceeding within. After the morning work was over the windows could generally be left wide open, as the sun almost always was streaming in, and we had bowls of the lovely wild flowers of Montenegro which began to peep out from the limestone crannies immediately the snow disappeared.

### Patients and Humbugs.

Except for a hurried round daily by sometimes a Dalmatian, sometimes a Dutch doctor, I was

* The first article appeared last week.

"monarch of all I surveyed." I soon established my authority by evicting a fat man who had been in charge before my arrival, but I could never find out what he did except try to steal the brandy. The committee had promised to remove him, but as it was a case of "words, not deeds," I seized the key of his room and commandeered his bed for a patient, and declined to re-admit him in spite of an appeal from the Archbishop himself. Montenegrin men do not work as a rule and the women are treated as inferior beings; it was funny to see how surprised my men were at having to obey me, but I had very little trouble with them. In my own mind I labelled my four wards as serious, chronic, exhaustion, and humbugs.

I divided the patients accordingly. New cases generally arrived towards evening, and I need hardly say that by the light of a dim oil lamp and under the grime of the battlefield it was easy to make a mistake, but it was more often that a serious case turned out to be nothing at all than the other way round. The thermometer would record a high temperature and the man look very ill; after a wash and rest and food he would look a different being next morning, and be able to leave the "serious" ward downstairs for one upstairs. There was such a continual changing of patients that I seldom could remember their "jaw-breaking" Slav names, and for me they remained only numbers, except when I had them in my care for some weeks. They were gay and talkative, and it was a continual worry to me that I could not understand Croatian. Fortunately the doctors all spoke French or German, as do the educated people throughout Montenegro.

I had to wage war against thieves—nothing was safe. Dusters and brushes, soap and towels, everything was coveted by the women, and many a cup or plate was carried off in one of their big striped bags, which they bore to their distant mountain homes after visiting their lords and masters. The men make interesting patients; they are cheerful, plucky, garrulous, brave, and idle.

Disciplining Convalescents.

They are entirely undisciplined, and once convalescent each man became a law unto himself, and in spite of rules laid down by the authorities the patients from the different hospitals left their precincts when it suited them; on market days the streets were full of lean and bandaged men in every variety of costume, from the frowzy dressing-gown to the gorgeous national costume of scarlet and blue and gold. They had great faith in the doctor and delighted in taking medicine, so to prevent jealousy everyone got something—a tonic if nothing else, or a liniment, for they firmly believed rheumatism could be rubbed away. One man who was admitted as possibly a case of enteric showed only symptoms of mental aberration, and the doctor was trying to find out if there were any asylum to which he might be sent when his mother urged that a monk might be brought from the monastery to exorcise the evil spirit. I therefore requisitioned one, and he came, tall and bearded, full of dignity, and clothed in gold vestments. He laid his stole over the face of the "possessed" and recited a service from an ancient book; all present fervently crossed themselves continuously, and the ceremony concluded with the burning of incense in the dustpan, which was taken to each person and wafted before him. The monk then withdrew, and with him the evil spirit, for from that moment Juro's brain was all right!

Medical Hospital, Cettigne, with Miss Callwell, Patients and Servants.

I had not a few malingerers, the pleasant life in hospital for convalescents, the daily saunter in the town, congenial company and regular meals, encouraged many to defer as long as possible their return to the front. But the news of the fall of Scutari soon revealed the humbugs; all their mysterious aches and pains fled, and they were amongst the foremost in marching all night about the streets, shouting, singing, and firing their revolvers which they had so carefully guarded under their pillows while they were in my little hospital.

## Figures and Tables

**Figure f1:**